# Effect of Hemostatic Agents on Shear Bond Strength of Orthodontic Eyelets Bonded with Conventional and Self-etch Adhesives

**DOI:** 10.30476/DENTJODS.2021.89677.1439

**Published:** 2022-06

**Authors:** Arian Hesam Arefi, Seyed Amir Hossein Mirhashemi, Tabassom Hooshmand, Mohammad Javad Kharazi Fard, Mohammad Sadegh Ahmad Akhoundi

**Affiliations:** 1 Dental Research Center, Zahedan University of Medical Sciences, Zahedan, Iran; 2 Dept. of Orthodontics, School of Dentistry, Tehran University of Medical Sciences, Tehran, Iran; 3 Dept. of Dental Biomaterials, School of Dentistry, Research Center for Science and Technology in Medicine, Tehran University of Medical Sciences, Tehran, Iran; 4 Statistical Advisor, Dental Research Center, Dentistry Research Institute, Tehran University of Medical Sciences, Tehran, Iran; 5 Laser Research Center of Dentistry, Dentistry Research Institute, Dept. of Orthodontics, Tehran University of Medical Sciences, Tehran, Iran

**Keywords:** Hemostatics, Shear strength, Orthodontic Brackets, Adhesives, Dental Cements

## Abstract

**Statement of the Problem:**

The risk of debonding of the orthodontic eyelets due to blood contamination from the impacted tooth is a clinical problem that orthodontists face routinely.

**Purpose:**

This study aimed to assess the effect of hemostatic agents (Viscostat clear and Astringedent X) on shear bond strength (SBS) of orthodontic eyelets bonded with conventional (Transbond XT) and universal adhesive (GC-G Premio bond) adhesives.

**Materials and Method:**

In this experimental study, 84 freshly extracted bovine lower incisors were randomly divided into 7 groups namely TBXT, Blood, SEP, VISCO + TBXT, VISCO + SEP, ASTX + TBXT, and ASTX + SEP groups. In the TBXT group, teeth were etched with phosphoric acid gel, and then, the Transbond XT primer was applied and eyelets were bonded with Transbond XT. In the blood group, first, contamination with blood was done followed by the same procedure as TBXT. In other groups, the teeth were etched with phosphoric acid and based on their group allocation, Transbond XT primer (in TBXT groups), GC-G Premio Bond (in SEP groups), ViscoStat Clear gel (in VISCO groups), or Astringedent X (in ASTX groups) were applied. The SBS was measured for each group and the adhesive remnant index (ARI) was determined.

**Results:**

The mean SBS was 20.79 MPa in VISCO+SEP group, 19.2 MPa in the TBXT group, 16.06 MPa in the SEP group, 15.43 MPa in VISCO+TBXT group, 9.39 MPa in AST-X+TBXT group, and 1.62 MPa in the blood group. The SBS of ASTX+SEP, blood and TBXT+ASTX groups had significant differences from the SBS of the control group (p< 0.05). The ARI score of 0 had the highest frequency in the blood group.

**Conclusion:**

Due to the high coagulative effect of Astringedent X and acceptable SBS of Astringedent X in combination with GC-G Premio bond, this combination can be recommended for exposure of impacted teeth that are at high risk of blood contamination.

## Introduction

An impacted tooth is a permanent tooth whose more than three-quarters of the root is formed and its self-eruption in a reasonable time is unexpected. Two different methods are applied in the clinics for the exposure of impacted teeth including close surgical exposure and open surgical exposure [ 1 ]. For bonding an attachment to the impacted teeth in the field of surgery and achieveng a clinically acceptable SBS of attachments, maintaining appropriate hemostasis during bonding is crucial.

Enamel bonding, which is the key procedure in either restorative or esthetic treatments, has been always challenging [ 2 ]. Conventionally, etch and rinse adhesives are used as the most popular adhesion approach in dentistry, but today, they are somehow substituted by self-etch adhesives for their more convenient handling and efficiency in bonding to enamel [ 3 ]. However, the new self-etching approaches have some disadvantages too. For example, the acids with higher pH used in such adhesives provide less enamel demineralization and weaker enamel bonds compared to phosphoric acid that is used in conventional etch and rinse approaches [ 4 ]. The bond-strengthening effect of the acid etching method is following the hydroxyapatite dissolution that creates regular microporosities, which increase the surface area for adhesion [ 5 ]. In self-etch adhesives, on the other hand, the acidic functional monomers interact with the mineral component of enamel providing the etching function [ 3 ]. In addition, the inter-prismatic acid etching pattern is deep in conventional adhesion approaches, while this pattern in self-etching techniques is absent to moderate. However, the enamel bond strength in these systems stays acceptable, regardless of their minimal acid attack on enamel [ 4 , 6 ]. Accordingly, Brackett *et al.* [ 7 ] claim no essential correlation between the bond strength and deep inter-prismatic acid attack. Whereas, Dalton *et al.* [ 8 ] addressed lower bond strength to enamel for self-etching systems compared to the conventional systems. Therefore, the efficacy of using phosphoric acid before self-etching approaches has been suggested to increase the retentive strength of self-etching adhesives [ 4 ].

In the new adhesion approaches, self-etching primers with less application time and no need for a completely dry surface, namely wet bonding, are used for bonding attachments [ 9 ].

Nowadays, contamination with blood and detachment of orthodontic attachment from impacted tooth surface is a clinical problem that results in more treatment duration, additional charges for the patient, and difficulties for both clinician and patient. Hemostasis is a complicated process with three major phases including vasoconstriction, platelet plug formation, and coagulation (in secondary hemostasis). Hemostatic agents increase hemostasis capacity either mechanically or by stimulating the coagulation cascade [ 10 ]. Chemical hemostatic agents are categorized as Class I (vasoconstrictors, adrenergic) and Class II (hemostatic agents, Astringedents) groups. Astringedents such as aluminum chloride, Alum (aluminum potassium sulfate), and zinc chloride sediment proteins on the surface of the mucosa and its mechanical strengthening. Stypics such as ferric sulfate and ferric chloride are concentrated forms of Astringedent and result in surface and local coagulation [ 11 ]. Astringedent X (Ultradent Products Inc., South Jordan, Utah, USA) is composed of 12.5% iron with an equal presence of ferric sulfate and ferric subsulfate. ViscoStat Clear (Ultradent Products Inc., South Jordan, Utah, USA) is composed of 25% aluminum chloride. Hemostatic agents with more acidity are more effective in coagulation but they are harmful to tissues [ 12 ].

Because of the lack of studies on these new hemostatic agents and new self-etch primer effects on shear bond strength (SBS) of orthodontic attachments, we decided to conduct this study. Accordingly, the current study aimed to determine the effect of hemostatic agents (Viscostat clear and Astringedent X) and the Universal adhesive (GC-G Premio bond) in comparison with a conventional bonding agent (Transbond XT) in bonding the orthodontic eyelets.

## Materials and Method

### Study design and experimental groups

According to Oksayan *et al.* [ 13 ] and using one-way analysis of variance (ANOVA), the least sample size was 12 samples in each group. This experimental study was conducted *in vitro* using 84 freshly extracted bovine lower incisors. At first, all tissue that remained around the root surface of teeth was mechanically removed, and then, teeth were washed under running tap water. For later usages, they were stored in distilled water. Then, teeth were randomly divided into seven groups (n=12) including TBXT group, blood group, SEP group, VISCO + TBXT group, VISCO + SEP group, ASTX + TBXT group, and ASTX + SEP group. Before removing, all tooth surfaces were cleaned with toothpaste, fluoride-free pumice, and water for 10 seconds. After every 5 times, the prophylaxis of the rubber cap was changed to ensure its proper functioning. The teeth were then fixed on a wax plate and then, the buccal surface of the teeth was examined for any enamel failure by the light-pass method using a stereomicroscope (Konix-120, Germany) at a magnification of 10X. Any enamel cracks and structural failures were confirmed. In the TBXT group, teeth were etched with phosphoric acid gel for 20 seconds and rinsed for 20 seconds, then, Transbond XT primer was applied and light-cured for 10 seconds. In the blood group, teeth surfaces were etched for 20 seconds and rinsed for 20 seconds. Then, they were contaminated with fresh blood of the author immediately after donating using a syringe and needle and the Transbond XT primer was applied and light-cured for 10 seconds. In this group, no type of anticoagulant was used and contamination with the blood sample was immediately performed after obtaining it. In the SEP group, teeth were etched with phosphoric acid for 20 seconds, and then, GC-G Premio bond (Japan, GC Corporation) was applied on the surface and light-cured for 10 seconds. In the VISCO + TBXT group, teeth were etched with phosphoric acid for 20 seconds, then, ViscoStat Clear (Ultradent, South Jordan, Utah, ABD) was applied for 2 minutes using a special syringe and tip, then, rinsed for 30 seconds. After, the Transbond XT primer was applied and light-cured for 10 seconds. In the VISCO + SEP group, teeth were etched with phosphoric acid for 20 seconds, then, ViscoStat Clear was applied for 2 minutes using a special syringe and tip and rinsed for 30 seconds. Then, the GC-G Premio bonds were applied and light-cured for 10 seconds. In the ASTX + TBXT group, teeth were etched with phosphoric acid for 20 seconds, and then, Astringedent X (Ultradent, South Jordan, Utah, ABD) was applied for 2 minutes using a special syringe and tip and rinsed for 30 seconds. After, the Transbond XT primer was applied and light-cured for 10 seconds. In the ASTX + SEP group, teeth were etched with phosphoric acid for 20 seconds, then, Astringedent X was applied for 2 minutes using a special syringe and tip, rinsed for 30 seconds, and GC-G Premio bond was applied and cured with light for 10 seconds. In all groups, the eyelets (Henry Schein Orthodontics, Melville, N.Y, USA) were bonded on the middle of the crowns with Transbond XT adhesive. After mounting, the teeth were thermocycling and SBS was measured by a universal testing machine. The adhesive remnant index (ARI) was also measured.

### SBS

After bonding, all specimens were stored in distilled water at 37±1ºC for 48 hours. Before testing, they were also thermal-cycled 3000 times between 5 ºC and 55 ºC. Samples were jigged using acrylic blocks. A stainless steel wire (0.2 mm diameter) was used to connect the samples to the jig. A 1mm/min force was applied on the samples until fracture happened and the maximum-tolerated force was recorded in MPa. Then, the mode of failure (adhesives, cohesive, and mixed) of specimens was determined using a Stereomicroscope (40 × magnification). The bond strength was evaluated using a universal testing machine (Zwick Roell, Ulm, Germany) in which the force of the device crosshead (1 mm/min) was applied on samples (N/mm^2^), and the strength was calculated in MPa with this formula: Shear bond strength (MPa) = debonding force (N)/surface area of the eyelet (mm^2^) .

The surface area of eyelets was 14.14mm^2^, according to the factory information. Bond strength was measured with a shear test, and failure modes were assessed. For evaluating the ARI, the buccal surface of each tooth was observed by a stereomicroscope (Konix-120, Germany) with 10× magnification and scored according to Artun and Bergland criteria [ 14 ].

### Statistical analysis

The obtained data were analyzed by one-way analysis of variance (ANOVA) using SPSS software version 22.0 (SPSS, Inc., Chicago, IL, USA). For showing the main effect of treatments, means were compared by Tukey-Kramer test. Kruskal-Wallis test was used for the analysis of ARI data. A p< 0.05 was considered statistically significant.

## Results

The shear bond strengths of orthodontic eyelets bonded using the conventional and self-etch adhesives are represented in Table 1. Accordingly, the resulted SBS values were 20.79 MPa for the VISCO + SEP group, 19.2 MPa for the TBXT group, 16.06MPa for the SEP group, 15.43MPa for the VISCO+TBXT group, 9.39MPa for the ASTX+TBXT group and 1.62MPa±1.74 for the blood group that showed the least SBS between all groups.

**Table 1:** Shear bond strength (mean±SD) of orthodontic eyelets bonded with conventional and self-etch adhesives

Groups	N	Minimum	Maximum	Mean	Std. Deviation
BLOOD	12	0.00	5.12	1.6267	1.74413
ASTX+SEP	12	1.91	16.43	11.0358	4.27160
VISCO+TBXT	12	4.10	31.73	15.4300	8.49315
SEP	12	3.03	27.82	16.0667	6.29525
TBXT	12	16.76	21.87	19.2092	1.79920
VISCO+SEP	12	7.01	31.31	20.7958	6.21683
ASTX+TBXT	12	4.13	16.44	9.3933	3.71543

ASTX: Astringedent X, SEP: Self -etch primer, TBXT: Transbond XT, VISCO: ViscoStat Clear

**Figure 1:**
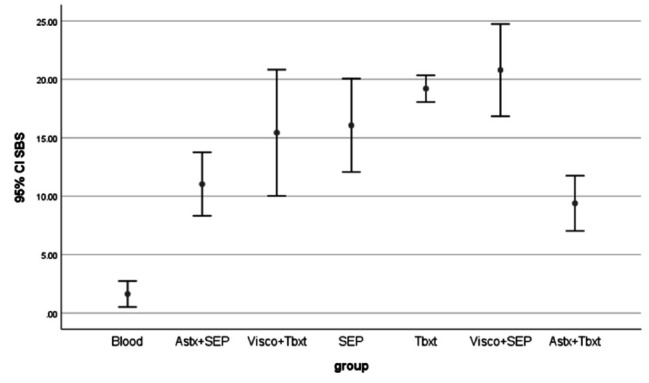
Mean shear bond strength and 95% CI of orthodontic eyelets bonded with conventional and self-etch adhesives

According to Table 2, the control group (TBXT) showed a statistically significant difference with ASTX+SEP (*p*= 0.000), blood (*p*= 0.000) and TBXT+ASTX (*p*= 0.000) groups, and the difference of SBS between control and VISCO+TBXT, SEP and VISCO+SEP groups was not statistically significant (Figure 1).

**Table 2:** Pairwise comparisons of shear bond strength of orthodontic eyelets bonded with conventional and self-etch adhesives

Group	*p* Value	Group	*p* Value
BLOOD vs. ASTX+SEP	0.49	ASTX+SEP vs. VISCO+TBXT	0.018
BLOOD vs. SEP	0.29	SEP vs. ASTX+TBXT	0.241
BLOOD vs. ASTX+TBXT	0.027	SEP vs. VISCO+SEP	0.068
BLOOD vs. VISCO+SEP	0.004	SEP vs. TBXT	0.056
BLOOD vs. TBXT	0.003	SEP vs. VISCO+TBXT	0.044
BLOOD vs. VISCO+ TBXT	0.002	ASTX+TBXT vs. VISCO+SEP	0.51
ASTX+SEP Vs. SEP	0.724	ASTX+TBXT vs. TBXT	0.41
ASTX+SEP Vs. ASTX+ TBXT	0.172	ASTX+TBXT vs. VISCO+TBXT	0.40
ASTX+SEP vs. VISCO+SEP	0.029	VISCO+SEP vs. TBXT	0.93
ASTX+SEP vs. TBXT	0.024	VISCO+SEP vs. VISCO+TBXT	0.85
TBXT vs. VISCO+TBXT	0.92		

ASTX: Astringedent X, SEP: Self -etch primer, TBXT: Transbond XT, VISCO: ViscoStat Clear

Based on Table 3, the blood group showed the most prevalence for zero grade of ARI index and ARI Index score 1 was the most prevalent score between different groups. Table 4  shows pairwise comparisons of the ARI Index. Only blood group ARI showed a statistically significant difference with VISCO+SEP (*p*= 0.004), TBXT (*p*= 0.003) and TBXT+VISCO (*p*= 0.002) groups.

**Table 3:** Prevalence of ARI different scores between study groups

			ARI				Total
			0	1	2	3	
Group	BLOOD	Count	5	6	1	0	12
		% within group	41.7%	50.0%	8.3%	0.0%	100.0%
	ASTX+SEP	Count	2	10	0	0	12
		% within group	16.7%	83.3%	0.0%	0.0%	100.0%
	VISCO+TBXT	Count	1	6	3	2	12
		% within group	8.3%	50.0%	25.0%	16.7%	100.0%
	SEP	Count	1	11	0	0	12
		% within group	8.3%	91.7%	0.0%	0.0%	100.0%
	TBXT	Count	0	8	3	1	12
		% within group	0.0%	66.7%	25.0%	8.3%	100.0%
	VISCO +SEP	Count	0	8	4	0	12
		% within group	0.0%	66.7%	33.3%	0.0%	100.0%
	ASTX+TBXT	Count	0	10	1	1	12
		% within group	0.0%	83.3%	8.3%	8.3%	100.0%
Total		Count	9	59	12	4	84
		% within group	10.7%	70.2%	14.3%	4.8%	100.0%

ASTX: Astringedent X, SEP: Self -etch primer, TBXT: Transbond XT, VISCO: ViscoStat Clear

**Table 4:** Pairwise comparisons of ARI Index of orthodontic eyelets bonded with conventional and self-etch adhesives

Groups	*p* Value
BLOOD & ASTX+SEP	0.490
BLOOD & SEP	0.297
BLOOD & ASTX +TBXT	0.027
BLOOD & VISCO+SEP	0.004
BLOOD & TBXT	0.003
BLOOD & VISCO+TBXT	0.002
ASTX+SEP & SEP	0.724
ASTX+SEP & ASTX +TBXT	0.127
ASTX+SEP & VISCO+SEP	0.029
ASTX+SEP & TBXT	0.024
ASTX+SEP & VISCO+TBXT	0.018
SEP & ASTX+TBXT	0.241
SEP & VISCO+SEP	0.068
SEP & TBXT	0.056
SEP & VISCO+TBXT	0.044
ASTX+TBXT & VISCO+SEP	0.513
ASTX+TBXT & TBXT	0.416
ASTX+TBXT & VISCO+TBXT	0.403
VISCO+SEP & TBXT	0.934
VISCO+SEP & VISCO+TBXT	0.856
TBXT & VISCO+TBXT	0.921

ASTX: Astringedent X, SEP: Self -etch primer, TBXT: Transbond XT, VISCO: ViscoStat Clear

## Discussion

The proper bond between the enamel and bracket is a requisite for an efficient orthodontic treatment [ 15 ]. Favorable SBS should be able to withstand the oral and occlusal forces during treatment, while the bracket should be also easily debonded without harming the enamel at the end of treatment [ 16 ]. The present findings showed acceptable SBS means for VISCO+SEP (20.79MPa), TBXT (19.2MPa), SEP (16.06 MPa), VISCO+TBXT (15.43MPa), ASTX+TBXT (9.39 MPa) and blood (1.62 MPa) groups. There was a significant difference in the SBS of ASTX+SEP, blood and TBXT +ASTX groups compared to the control group (*p*< 0.05). The standard deviations showed a high tendency to individual variation. Either of the self-etching systems with strong to mild pH could not etch enamel as deeply as the phosphoric acid. This is while the shallow etching patterns are discussed to provide a weak bonding to the enamel [ 17 ]. In the same regard, the acidity level of the acidic primers or self-etching adhesive solutions is the main determinative of the demineralization function of self-etching systems [ 17 ]. Although one study reports a lower adhesion to the ground enamel in self-etching approaches in comparison with the conditioning of the ground enamel by phosphoric acid [ 18 ], the self-etching systems are still suggested as efficient alternatives to the phosphoric acid-utilizing systems. This is while the etching aggressiveness of self- etching primers does not contribute to their efficacy in cases of the ungrounded enamel.

The comparative study of Khanehmasjedi *et al.* [ 19 ] on the effect of dry conditions and contamination with saliva on the SBS of metallic brackets showed that saliva contamination reduces the bond strength of assuring bonding agent, and using the single bond and assure bonding agents do not provide enough bond strength of brackets for tooth structures. Rix *et al.* [ 20 ] also showed that using Assure universal bonding resin makes no difference in the SBS of brackets to enamel under saliva-contamination conditions. Whereas, in another similar study, Eslami *et al.* [ 16 ] bonded the stainless steel brackets to enamel using Assure adhesive resin and achieved an adequate bond strength in both dry (14.18 MPa) and saliva contamination (13.32 MPa) conditions. The SBS for clinically applicable orthodontic brackets ranges from 5.9 to 7.8 MPa [ 17 ]. Therefore, the bonding strengths achieved by both single bond and assure bonding agents used in the Eslami *et al.*’s [ 16 ] under dry and wet conditions were adequate for tooth structures. In addition, Kanca *et al.* [ 21 ] used a dentin-bonding agent that provided comparable bond strength in both dry and wet enamel conditions. However, their results showed slightly higher bond strength on dry enamel surfaces. Despite this, the findings of Wakefield *et al.* [ 22 ] indicated that using dentin-bonding agents could minimize the effect of moisture presenting on the enamel surface on the bond strength.

In the present study, the Gc-G Premio bond showed insignificant SBS in comparison to the Transbond XT primer. In Bishara *et al.*’s [ 23 ] study, Prompt L-pop showed lower significant SBS in comparison to Transbond XT primer. Although the Prompt L-pop is an aggressive self-etch primer and has more etching depth in enamel, the Gc-G Premio bond showed insignificant SBS in comparison to the Transbond XT primer. The reason for this difference can be due to differences in the solvent and monomer composition of these two bonding agents.

Trakyali *et al.* [ 24 ] used bovine incisor teeth, ankaferd blood stopper (ABS) hemostatic agent, and blood for contamination. Similar to the present study, the control group of their study was treated with Transbond XT adhesive. Their control group showed maximum SBS (19 MPa) that was close to that of the present study (19.2 MPa). In addition, the blood group of Trakyali *et al.*’s study (4.5 MPa) showed more SBS in comparison to our blood group (1.62 MPa). In the current study, samples of the blood group were contaminated with the etched surface of enamel using fresh blood in minimum time and no anticoagulant agents were used because they could interfere with blood. Astringedent X showed a similar decrease in SBS similar to the ABS hemostatic agent. It could be a result of the molecular size of these hemostatic agents and the chemical interactions between hemostatic agents and hydroxyapatite of the etched surface [ 25 ].

Gungur *et al.* [ 26 ] used impacted third molars, blood, and ABS for contamination of enamel surface before etching. They used light bonds as adhesive; however, the comparison between studies is unlikely because of the substantial differences in methods. According to the current findings and those of Güngör *et al.* [ 26 ], SBS is decreased in case of contamination either before or after etching. 

Oksayan *et al.* [ 13 ] used the human premolar teeth, Transbond XT as the control group, blood, and ABS and epinephrine as hemostatic agents. Based on their results, the SBS of the blood group (2MPa) was close to that of the present study (1.62 MPa). ABS and epinephrine like Astringedent X decreased the SBS.

Karabekiroglu *et al.*’s [ 27 ] study included both *in vitro* and in vivo parts. In the *in vitro* part, they used human premolar teeth and, in all groups, blood contamination was done. In their study, contamination with blood and hemostatic agents (Vistostat, Epinephrine, and H2O2) was performed before etching which is unlike the current study. This study did not use any self-etch primer in study groups. Similar to the present study, hemostatic agents decreased SBS but in an acceptable clinical range (5.9-8.7MPa) according to Reynolds and Von Fraunhofer [ 28 ]. In the in vivo part of this study, enamel surfaces were also contaminated before etching. Because bleeding can occur at different stages (before or after etching, or after administration of bonding agent), it is acceptable to study SBS with blood or hemostatic agent contamination in these different phases.

Currently, a combination of phosphoric acid (37%) and Transbond XT is considered the most common protocol for orthodontists in their experimental studies [ 29 ]. Grubisa *et al.* [ 30 ] compared the SBS produced by self-etching primer plus Transbond XT composite resin, phosphoric acid plus Transbond XT composite resin and phosphoric acid plus Enlight bonding composite resin. They reported no significant difference among all the aforementioned combinations.

In terms of mechanism, the self-etching dental adhesion systems effectively bond to the enamel surfaces through the nano-retentive interlocking between crystallites and the thin hybridized complex of adhesive resin formed in the enamel, despite the lower bond strengths presented by some self-etching systems [ 17 ]. In this regard, the acidic resin monomers within the self-etch adhesives eliminate the need for a prior etching step on dental substrates by phosphoric acid. It is noteworthy that the resin monomer composition, water content, and acidity of different self-etch adhesives are variable [ 31 ]. On the other hand, in routine orthodontic procedures, a safe debonding of brackets is more important than maximum bond strength [ 32 ]. According to factory Astringedent X is a stronger hemostat agent than Viscostat clear .Moreover, it is reported that self-etch primers like Gc-G Premio bond combine two crucial steps of the bonding process and perform better than total-etch systems in wet fields, on the other hand, the SBS of this combination is within the acceptable clinical range [ 28 ]. Due to the high coagulative effect of Astringedent X and acceptable SBS of Astringedent X and GC-G Premio bond combination, they can be recommended for exposure of impacted teeth at high blood contamination risk.

Considering the reliability of ARI scores for determining the bond failure location in enamel, adhesive, and bracket base in various studies, Khanehmasjedi *et al.* [ 19 ] surveyed the frequencies of ARI scores between different study groups and found no significant difference. This score evaluates the remained amount of composite resin on enamel surfaces. In the current study, the blood group showed that the most prevalent ARI index equals zero. This results from very low SBS of the blood group and debonds of the adhesive from the tooth surface. Only the ARI in Blood group showed a statistically significant difference with VISCO + SEP, TBXT, and TBXT + VISCO groups. Also, SBS of the Blood group and the TBXT group showed a statistically significant difference.

This *in vitro* study had potential limitations including experimental design, use of bovine teeth; there are different hemostatic agents like Ankaferd blood stopper, Epinephrine, and Astringedent that we could not use in our groups because of the limitation of study samples.

## Conclusion

Using Viscostat clear in addition to GC-G Premio bond had probably the highest SBS, and this combination is recommended for exposure of impacted teeth in normal condition. Due to the high coagulative effect of Astringedent X and acceptable SBS of Astringedent X and Gc-G Premio bond combination, they can be recommended for exposure of impacted teeth at high blood contamination risk and they can be used for patients at high risk of bleeding, including hemophilic patients.

## Acknowledgement

This study was funded and supported by Dental Research Center, Dentistry Research Institute, Tehran University of Medical Sciences, Grant No: 97-03-70-40188.

## Conflict of Interest

None declared.
